# Effectiveness of the One-Minute Paper as a Continuous Internal Assessment Tool for Learners and Teachers

**DOI:** 10.7759/cureus.94750

**Published:** 2025-10-16

**Authors:** Dina Raja, Harekrishna Nath, Rika Engtipi, Nishanta Thakuria, Devyashree Medhi, Divya Daimari, Adarshlata Singh, Putul Mahanta

**Affiliations:** 1 Microbiology, Dhubri Medical College and Hospital, Dhubri, IND; 2 Pharmacology and Therapeutics, Dhubri Medical College and Hospital, Dhubri, IND; 3 Dermatology, Venereology and Leprosy, Jawaharlal Nehru Medical College, Datta Meghe Institute of Higher Education and Research, Wardha, IND; 4 Forensic Medicine and Toxicology, Nalbari Medical College, Nalbari, IND

**Keywords:** competency-based medical education, formative assessment, national medical commission, one-minute paper, student learning

## Abstract

Background

An assessment tool is necessary to help medical students adequately grasp their learning and to allow them to ask, discuss, and answer questions about the material being taught. The One-Minute Paper (OMP) is a rapid and effective formative assessment tool used in educational settings to enhance learning and teaching. The present interventional study aims to assess the effectiveness of the OMP as a continuous assessment tool for teachers and learners and to investigate the perceptions of learners and faculty regarding OMP as a continuous internal assessment tool.

Methodology

This prospective, interventional study, based on online questionnaire feedback, was conducted at Dhubri Medical College and Hospital (DMCH), Dhubri, from February 2024 to July 2024. The study participants were Phase 2 MBBS students from DMCH, Dhubri, Assam. A sample of 100 students was selected using a convenience sampling method. Google Forms was created with questions or prompts related to the content covered in the class or lesson, and the link was shared with learners at the end of the session, instructing them to complete the form within one minute. Descriptive statistical methods were used to analyze the data.

Results

Of the 100 Phase II MBBS students who participated in the study, 83-90 participants in the five lectures provided complete responses on all components of the OMP questionnaire. In total, 425 completed replies were gathered from the five groups, among whom 253 (59.5%) rated the sessions as highly useful. An average of 97.4% (n = 414/425) reported that all learning goals covered in class were clearly described. Additionally, 97.6% (n = 415/425) reported that the provided topics were useful and improved their learning. Participants sought greater explanations of laboratory testing methods. Overall, 45.0% (n = 191) of participants appreciated the excellent performance of OMP, while another 32.0% (n = 136) marked it as a successful initiative.

Conclusions

Most participants believed that the OMP is a suitable technique for assessing lessons learnt in lectures. The OMP was useful for assessing the utility of the concepts taught and providing educators with information on the efficacy of their teaching strategies. Input from previous sessions helped them identify knowledge gaps and refine their procedures.

## Introduction

The National Medical Commission (NMC) has introduced Competency-Based Medical Education (CBME) for the Undergraduate Medical Curriculum in India, aiming to raise the standards of Indian medical graduates and achieve the goal of “Health for All” [1]. As an “outcome-based” approach, CBME emphasizes shared accountability with students, uses student-centered teaching strategies, and incorporates formative evaluation as a crucial part of the educational process. It focuses on medical graduates’ attitudes, strengthening their clinical skills, and enhancing their knowledge of medicine [2]. Enhancing comprehension of the subject matter covered in lectures is the goal of all medical educators. Continuous assessment helps the teaching faculty improve their methods based on learners’ feedback. In graduate medical education, one obstacle to methodically evaluating feedback regarding the structure or content of teaching is the time required to gather and examine feedback data. Internal assessment (IA) is a critical component of medical education that helps evaluate various competencies through formative and summative assessments. Summative assessments evaluate students’ learning at the end of a professional course; therefore, they provide limited feedback. At the same time, formative assessments help monitor student learning and provide ongoing feedback that instructors can use to enhance their teaching and that students can use to improve their learning. To provide adequate information on medical students’ learning and to allow them to ask, discuss, and answer questions about the material being taught, a variety of assessment instruments are used.

The One-Minute Paper (OMP) is a quick and effective formative assessment tool used in educational settings to enhance learning and teaching. It is an effortless and efficient method for engaging students and providing immediate feedback on their learning [3]. Furthermore, it supplies mediator educators with information about how well their approaches are received. The OMP involves asking students to spend a minute at the end of a class session writing down brief responses to questions such as “What was the most important thing you learnt today?” or “What questions do you still have?” This method provides immediate feedback to both students and instructors, facilitating reflective learning and continuous improvement in teaching strategies [4]. The OMP serves as a powerful reflective practice for learners by encouraging them to consolidate their learning by summarizing key points, which aids in retention and understanding of the material. Moreover, the OMP helps students develop metacognitive skills by making them aware of their learning processes and identifying areas that need further clarification [5]. From teachers’ perspective, the OMP provides immediate feedback on students’ understanding and misconceptions, empowering them to close learning gaps and adjust their teaching strategies promptly. By regularly implementing the OMP, teachers can gauge the effectiveness of their teaching strategies and make data-driven decisions to enhance their pedagogy [6]. The OMP is a flexible and effective tool for continuous internal evaluation. Students benefit from it by encouraging introspection and self-evaluation, while teachers receive valuable input to improve their methods. Thus, OMP facilitates the development of a dynamic and adaptable learning environment that encourages continuous progress for both teachers and students.

Research indicates that using OMP has numerous benefits and is also cost-effective. However, medical educators have not extensively adopted this potentially valuable classroom assessment tool. Therefore, the current interventional study was conducted among Phase II MBBS students at a medical college hospital in the northeastern region of India. The specific objectives of the study were (1) to assess the effectiveness of the OMP as a continuous assessment tool for teachers and learners in terms of perceived usefulness, learner satisfaction, or measurable improvement in learning performance; and (2) to analyze the perceptions of learners and faculty regarding OMP as a continuous internal assessment tool.

The primary outcome was students’ self-reported perception of learning improvement and session usefulness. The secondary outcomes included the number and type of learning gaps identified via OMP responses. Therefore, the present study aimed to evaluate the effectiveness of the OMP as a formative assessment tool by measuring its impact on learners’ perceived understanding of lecture content and its utility for guiding teachers in modifying subsequent sessions.

## Materials and methods

This study was a prospective, interventional study based on online questionnaire feedback conducted at Dhubri Medical College and Hospital (DMCH), Dhubri. The study lasted six months, from February 2024 to July 2024. The study participants were Phase II MBBS students at DMCH, Dhubri, Assam. A sample of 100 students was selected using convenience sampling. Prior informed consent for voluntary participation was obtained from each participating student, ensuring full confidentiality. The ethical clearance was obtained from the Institutional Ethics Committee of DMCH, Dhubri (approval number: DMCH/IEC/2024/19).

Inclusion and exclusion criteria

We included Phase II MBBS learners who are willing to participate in the study and provided informed consent. Phase II MBBS students who were absent or unwilling to participate were excluded. The students’ participation was voluntary. Students were given one minute at the end of the theory lecture sessions to provide feedback via a Google Forms questionnaire. The OMP assessment was conducted immediately after each session, and students were instructed to respond within one minute. The five theory classes were conducted on May 11, 2024, May 15, 2024, June 07, 2024, July 04, 2024, and July 17, 2024.

Data collection tool

Data were collected using a Google Forms questionnaire (Appendices) for the OMP assessment, which was validated through a pilot study involving 15 students and subsequently peer-reviewed and certified by members of the Medical Education Unit. The resources required for the study included access to Google Forms via a Google account, computers, tablets, or smartphones for learners and teachers to use the online forms, and an internet connection for online access.

A Google Forms questionnaire was created with questions or prompts related to the class or lesson content. The Google Forms link (https://docs.google.com/spreadsheets/d/1n4CLlnyDABUzYWBIfP4lDu7y9DGeQQB07GyO4w4epQ8/edit?usp=drive_link) was shared with learners at the end of the session, with instructions to complete the form within one minute. The form was set to automatically collect responses and provide instant feedback to learners if needed. The responses collected were assessed for individual understanding and common areas of confusion; any knowledge gaps were identified and analyzed. The insights gained will be used to tailor future lessons and provide additional support to students as necessary. The lecture content aligned with the specific learning objectives, generating ideas and knowledge on the given topic.

The Google Forms questionnaire for the OMP assessment included initial information for each participant and seven questions on the lecture topic. The first, sixth, and seventh questions were student- and content-focused, related to understanding the subject matter, which would allow the faculty to readdress any areas that were less understood. The first question probed the participants to rate the overall usefulness of the session on a five-point rating scale ranging from no usefulness (1) to very high usefulness (5). Questions six and seven were open-ended, verbatim questions for participants to respond to based on the lecture session’s learning objectives. Question six focused on the most important things they learnt from the lecture, and Question seven asked the participants to specify various questions still on their minds related to the lecture topic. The responses were thematically analyzed.

The second, third, fourth, and fifth questions were included to help the faculty improve their understanding of students’ perceptions of their effectiveness as teachers, thereby allowing them to reflect on their approach and amend it to meet the needs of the student cohort better. Question two enquired whether all the learning objectives covered in the lecture were clearly explained. The third question prompted participants to share their perceptions about whether the lecture topic was effective and whether it enhanced their learning. In the fourth and fifth questions, participants were asked about their perceptions of the methods the lecturer would adopt to enhance learning. The eighth question was designed to gather feedback on the use of OMP. Responses were thematically analyzed, and unclear concepts were revisited in the next session.

Statistical analysis

Descriptive statistical methods were used to analyze the data. The data obtained from Google Forms was extracted and analyzed using Microsoft Excel (Microsoft Corp., Redmond, WA, USA) and SPSS version 22 (IBM Corp., Armonk, NY, USA). The percentages and frequencies were used to display categorical variables. The Mantel-Haenszel chi-square test was used to test for a linear trend in ratings across sessions. A p-value <0.05 was considered significant.

## Results

The responses of five OMPs were analyzed, and the nature of the responses was noted. Of the 100 Phase II MBBS students participating in the study, complete responses to all the components of the OMP questionnaire were given by 83-90 participants in the five lectures. Overall, 425 completed responses were obtained in the five classes, of which 273 (64.2%) were from male participants (Table 1).

**Table 1:** Gender distribution of the participants in each lecture session. The data have been represented as frequency (n) and percentage; total sample N = 425.

Lectures	Topic	Male (%)	Female (%)
Class 1 (n = 90)	Etiopathogenesis, laboratory diagnosis, and prevention of infections of the upper and lower respiratory tract	58 (64.4%)	32 (35.6%)
Class 2 (n = 87)	Microbial agents causing diarrhea and dysentery. The epidemiology, morphology, pathogenesis, clinical features, and diagnostic modalities of these agents	57 (65.5%)	30 (34.5%)
Class 3 (n = 83)	Etiopathogenesis, clinical course, and the laboratory diagnosis of meningitis	54 (65.1%)	29 (34.9%)
Class 4 (n = 82)	Etiopathogenesis and the laboratory diagnosis of infections of the genitourinary system	51 (62.2%)	31 (37.8%)
Class 5 (n = 83)	Etiopathogenesis and the laboratory diagnosis of sexually transmitted infections	53 (63.9%)	30 (36.1%)
Total (n = 425)		273 (64.2%)	152 (35.8%)

The first question of the Google Forms questionnaire asked the participants to rate the usefulness of the session on a five-point Likert scale. Of the 90 participants who attended the first session on upper and lower respiratory tract infections, only half (n = 46, 51.1%) reported that the session was highly useful. The students’ perceptions were more favorable in later sessions, with over 67% (n = 56/83) of the participants rating the fifth session on sexually transmitted infections as highly useful. Session 3, covering the topics of etiopathogenesis, clinical course, and laboratory diagnosis of meningitis, was rated as useful to highly useful by more than 95% (n = 79/83) of the participants. Of the 425 total responses, 253 respondents rated the sessions as highly useful, with an overall rating of 59.5%. The Mantel-Haenszel chi-square test revealed a significant linear trend in student ratings across sessions (chi-square value = 5.86; p = 0.015), confirming improvement in ratings across lectures (Table 2).

**Table 2:** Overall rating for the usefulness of the session. The data have been represented as frequency (n) and percentage; total sample N = 425. #: Mantel-Haenszel chi-square test for linear trend; ##: p-value <0.05 is considered significant.

Lectures	Overall rating					Chi-square for linear trend^#^	P-value^##^
	1	2	3	4	5		
Total (n = 425)	2 (0.5%)	3 (0.7%)	36 (8.5%)	131 (30.8%)	253 (59.5%)		
Class 1 (n = 90)	0 (0.0%)	2 (2.2%)	9 (10.0%)	33 (36.7%)	46 (51.1%)	5.86	0.015
Class 2 (n = 87)	1 (1.1%)	1 (1.1%)	10 (11.5%)	28 (32.2%)	47 (54.0%)		
Class 3 (n = 83)	0 (0.0%)	0 (0.0%)	4 (4.8%)	25 (30.1%)	54 (65.1%)		
Class 4 (n = 82)	1 (1.2%)	0 (0.0%)	7 (8.5%)	24 (29.3%)	50 (61.0%)		
Class 5 (n = 83)	0 (0.0%)	0 (0.0%)	6 (7.2%)	21 (25.3%)	56 (67.5%)		

Table 3 presents the participants’ responses to the second and third questions, which are categorized as “Yes” and “No.” For the second question, an average of 97.4% (n = 414/425) of the participants indicated that the classes clearly explained all the learning objectives. Furthermore, 97.6% (n = 415/425) of the participants responded that the topics covered were effective and enhanced their learning.

**Table 3:** Students’ perception toward the effectiveness of the lecture sessions. The data have been represented as frequency (n) and percentage; total sample N = 425.

Lectures	Question 2: Did the teacher clearly explain all the learning objectives covered in today’s class?	Question 3: Did the topic covered today effectively enhance your learning?
	Response: Yes	Response: Yes
Class 1 (n = 90)	88 (97.8%)	89 (98.9%)
Class 2 (n = 87)	85 (97.7%)	85 (97.7%)
Class 3 (n = 83)	81 (97.6%)	81 (97.6%)
Class 4 (n = 82)	78 (95.1%)	78 (95.1%)
Class 5 (n = 83)	82 (98.8%)	82 (98.8%)
Total (n = 425)	414 (97.4%)	415 (97.6%)

Overall, 145 (34.1%) respondents indicated that the faculty’s presentation style was the most effective and enhanced student learning, followed by interactive sessions (n = 144, 33.9%) and audio-visual content (n = 50, 11.8%). Almost one-tenth of the total responses (n = 42, 9.9%) indicated that students found no change in the faculty’s teaching approach to enhance their learning, particularly in the fourth session (n = 16/82, 19.5%) regarding infections of the genitourinary system (Table 4).

**Table 4:** Responses to the question: what did the faculty do today that was effective and enhanced your learning? The data have been represented as frequency (n) and percentage; total sample N = 425.

Lectures	Interactive session	Group activity	Audio-visual content	Feedback	Style of presentation	No change
Class 1 (n = 90)	30 (33.3%)	3 (3.3%)	10 (11.1%)	3 (3.3%)	38 (42.2%)	6 (6.7%)
Class 2 (n = 87)	33 (37.9%)	2 (2.3%)	10 (11.5%)	8 (9.2%)	27 (31.0%)	7 (8.0%)
Class 3 (n = 83)	25 (30.1%)	5 (6.0%)	8 (9.6%)	10 (12.0%)	26 (31.3%)	9 (10.8%)
Class 4 (n = 82)	23 (28.0%)	0 (0.0%)	11 (13.4%)	6 (7.3%)	26 (31.7%)	16 (19.5%)
Class 5 (n = 83)	33 (39.8%)	1 (1.2%)	11 (13.3%)	6 (7.2%)	28 (33.7%)	4 (4.8%)
Total (n = 425)	144 (33.9%)	11 (2.6%)	50 (11.8%)	33 (7.8%)	145 (34.1%)	42 (9.9%)

Of the 425 respondents, 221 (52%) did not want any change in the way classes are taken, while 15.3% (n = 65) wanted an increase in interactive sessions, and another 11% (n = 47) wanted an increase in audio-visual content. Additionally, 6.8% (n = 29) sought improvement in the presentation style (Table 5).

**Table 5:** Responses to the question: what can the faculty do to improve the effectiveness of the class and enhance students’ learning? The data have been represented as frequency (n) and percentage; total sample N = 425.

Lectures	Increase the interactive session	Increasing group activity	Increase audio-visual content	More feedback	Improve the style of presentation	No change	Others
Class 1 (n = 90)	15 (16.7%)	6 (6.7%)	10 (11.1%)	5 (5.5%)	8 (8.9%)	42 (46.7%)	4 (4.4%)
Class 2 (n = 87)	12 (13.8%)	7 (8.0%)	13 (14.9%)	5 (5.7)	5 (5.7)	40 (46.0)	5 (5.7%)
Class 3 (n = 83)	13 (15.7%)	4 (4.8%)	8 (9.6%)	4 (4.8%)	7 (8.4%)	46 (55.4%)	1 (1.2%)
Class 4 (n = 82)	13 (15.9%)	6 (7.3%)	8 (9.8%)	3 (3.7%)	6 (7.3%)	45 (54.9%)	1 (1.2%)
Class 5 (n = 83)	12 (14.5%)	5 (6.0%)	8 (9.6%)	6 (7.2%)	3 (3.6%)	48 (57.8%)	1 (1.2%)
Total (n = 425)	65 (15.3%)	28 (6.5%)	47 (11.0%)	23 (5.4%)	29 (6.8%)	221 (52.0%)	12 (2.8%)

The participants’ opinions on the most significant lessons they took away from the lectures are summarized in Table 6. In the first lecture session, the most important aspects of the talk reported were the respiratory tract infections (n = 78, 86.7%) and their pathophysiology (n = 67, 74.4%). During the second session, students reported primarily learning about the various agents of diarrhea (n = 72, 82.7%) and their pathogenesis (n = 59, 67.8%). Most participants in the third session prioritized learning about the agents that cause meningitis (n = 68, 81.9%) and their clinical characteristics and pathophysiology (n = 62, 74.7%). During the fourth session, students primarily learned about the genitourinary system (n = 63, 76.8%) and the pathogenesis of infections (n = 59, 71.9%). The fifth session was observed to be the most successful in delivering learning objectives, with over 90% (n = 77, 92.8%) of the participants learning about the causative agents of sexually transmitted infections, their clinical features, and pathogenesis (n = 68, 81.9%), as well as laboratory diagnosis methods (n = 68, 81.9%). We also observed that students learned about laboratory diagnosis methods less frequently in most classes.

**Table 6:** Responses to the question: what are the most important things learned from the lectures? (multiple responses). The data have been represented as frequency (n) and percentage.

Lectures	The most important things learned from the lectures	Frequency (%)
Class 1 (n = 90)	Infections of the respiratory tract: its transmission	78 (86.7%)
	Pathogenesis	67 (74.4%
	Lab diagnosis	52 (57.8%)
Class 2 (n = 87)	The different agents of diarrhoea	72 (82.7%)
	Pathogenesis	59 (67.8%)
	Management	51 (49.4%)
Class 3 (n = 83)	Agents causing meningitis	68 (81.9%)
	Clinical features and pathogenesis	62 (74.7%)
	Lab diagnosis and management	53 (63.85%)
Class 4 (n = 82)	Basic concepts of the genitourinary system	63 (76.8%)
	Agents causing infection and its pathogenesis	59 (71.9%)
	Lab diagnosis and management	48 (58.5%)
Class 5 (n = 83)	Agents causing sexually transmitted infections	77 (92.8%)
	Clinical features and pathogenesis	68 (81.9%)
	Lab diagnosis and management	68 (81.9%)
	Preventive measures	59 (71.1%)

More than 50% of the students had no questions about any of the lecture topics. *Escherichia coli* pathogenesis, pathogenesis of viral meningitis, horizontal and vertical transmission of sexually transmitted infections, and laboratory diagnosis methods were some of the areas where participants wanted more explanations. Some participants also suggested incorporating multiple-choice questions and key questions related to the topic (Table 7).

**Table 7:** Responses to the question: what questions do you still have in your mind? The data have been represented as frequency (n) and percentage.

Lectures	Question 8: What questions do you still have?	
	No questions	Specific responses
Class 1 (n = 90)	71 (78.7%)	Few students want to know the important questions and the approach to the topic for the examination
Class 2 (n = 87)	60 (68.9%)	Few students wanted fewer slides. Some of the students have doubts regarding *Escherichia coli* pathogenesis
Class 3 (n = 83)	61 (73.4%)	Few students had doubts about the lab diagnosis and pathogenesis of viral meningitis
Class 4 (n = 82)	69 (84.0%)	Only a few students had questions about lab diagnosis and pathogenesis. Some students requested that multiple-choice questions and clinical scenarios be added
Class 5 (n = 83)	52 (62.6%)	A few students had doubts regarding the horizontal and vertical transmission of sexually transmitted infections and the lab diagnosis

**Figure 1:**
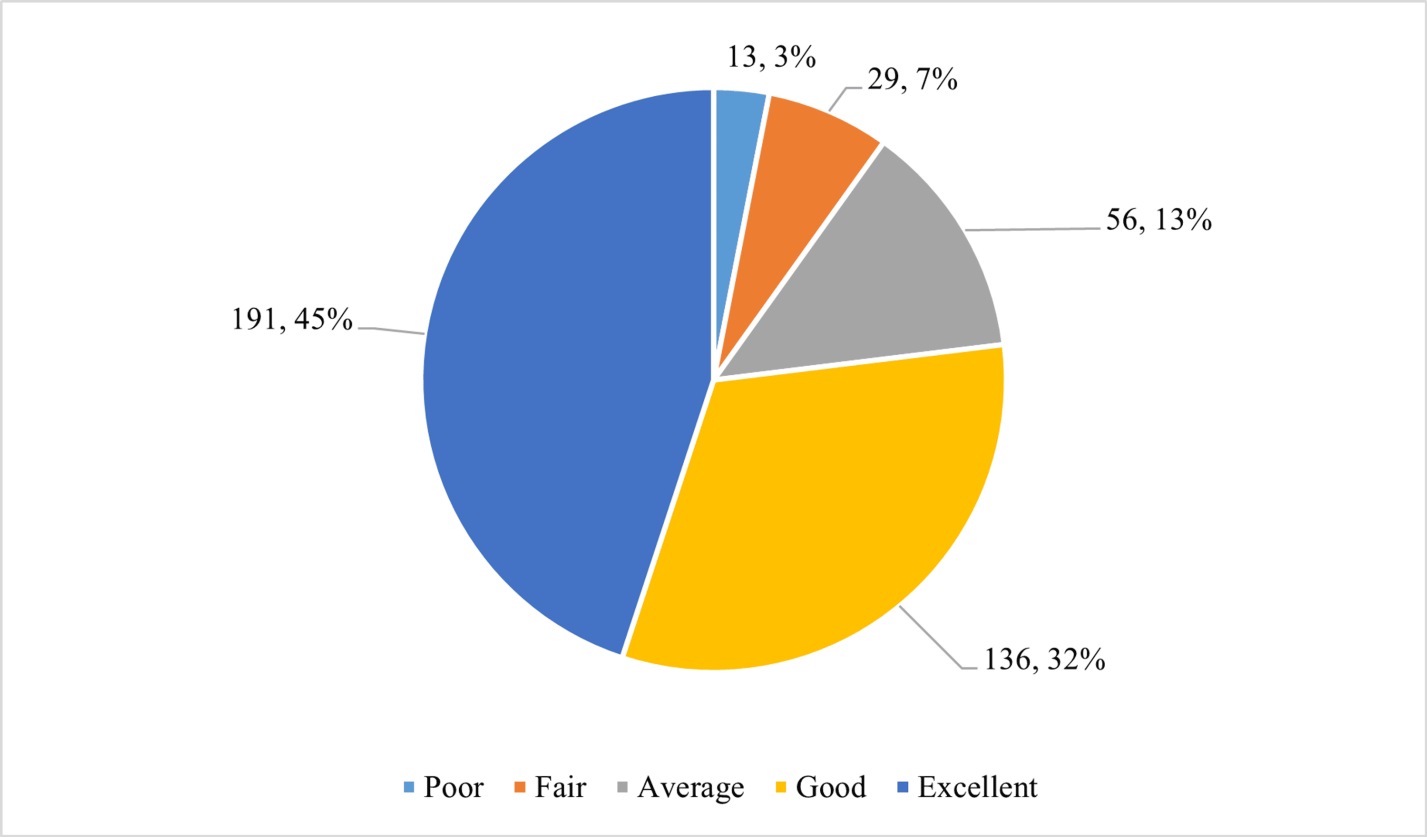
Students’ feedback on the One-Minute Paper tool (N = 425).

Figure 1 illustrates participants’ overall feedback on the use of the OMP tool for assessing lessons learned during the lecture sessions. Of the 425 respondents, 191 (45.0%) reported that taking OMP after each class was excellent.

## Discussion

In 2019, India’s NMC implemented CBME to increase the competency and practice-readiness of medical graduates by shifting medical education from a time-based model to one that emphasizes the development and demonstration of specific competencies in students [7]. When implementing a competency-based program, assessment is essential. Summative evaluation is conducted after the professional year for the traditional type of assessment in time-based medical education, which is primarily dependent on recall of factual information [8]. At the same time, the competency-based assessment process is ongoing throughout the year. It tracks students’ routine progress, including their efficiency, work ethic, disposition, and other academic pursuits. IA is a crucial part of medical education and serves as the basis for the CBME program. It helps evaluate different competencies. IA encompasses both formative and summative evaluations, and employing various techniques can enhance its efficacy. Within the IA framework, it is essential to provide regular feedback and corrective actions [9]. Every teacher of a subject and every competency should be involved in the process. It is necessary to establish a culture of regular feedback at the institute level. Prioritized corrective procedures should be made available to students who are unable to meet the skills and requirements in university examinations to improve their performance [10].

The OMP is a concise and useful formative evaluation instrument designed to enhance instruction and learning. In a short time, usually one minute, this dynamic evaluation method challenges students to explain what they understand about a subject. Its use encourages critical thinking, active participation, and reflection, enabling students to synthesize their knowledge by identifying important ideas and resolving ambiguities [11]. From the learners’ perspective, the OMP provides immediate feedback on their understanding and retention of material. In the present study, 59.5% (n = 253) of students rated the lecture sessions as highly useful, and 97.6% (n = 415) reported that the topics covered were effective and enhanced their learning. In today’s fast-paced learning environment, the brevity of the OMP ensures that students focus on the most important details, enhancing their ability to synthesize information quickly. Students are empowered to identify their learning gaps and ask questions about subjects they find difficult, thereby promoting a culture of ongoing learning and self-evaluation.

The OMP is a useful instrument for educators to assess classroom dynamics and the efficacy of teaching strategies. Teachers can identify common misconceptions, modify their teaching methods, and realign their lesson plans to better meet their students’ needs by examining student feedback. In the present study, students primarily believed that the presentation style (34.1%, n = 145) was the most effective in enhancing their learning, followed closely by interactive sessions (33.9%, n = 144). This feedback loop contributes to the overall improvement of teaching practices, creating a responsive educational environment. Overall, 52.0% (n = 221) did not want any change in the way they took the class. Further, 15.3% (n = 65) expressed a desire for more interactive sessions, and 11.0% (n = 47) for more audio-visual content. Session-wise data showed that the classes gradually improved as changes were made in response to student feedback after each class. However, we acknowledge that the evidence in the current study is based on self-reported data rather than objective performance outcomes. A recent study among 400 MBBS students and 11 faculty members covering 148 interactive lectures found that students considered the OMP useful for expressing doubts (73%), addressing least-understood ideas (86%), and increasing confidence in applying taught concepts (60%). The faculty praised the OMP as an effective formative and self-assessment tool for improving lecture quality and refining the curriculum [12].

It was also noted that students were more comfortable learning about the etiopathogenesis and clinical management of the various diseases covered in the lecture sessions. They were mostly doubtful about the laboratory diagnosis and management of those agents. Appropriate use of laboratory testing is crucial to providing patients with safe and efficient care. An inadequate understanding may lead to subpar case management, thereby increasing medical expenses. Laboratory testing instruction for undergraduate medical students is considered insufficient. Research suggests that, to improve clinical decision-making and ensure optimal modern medical treatment, medical curricula should prioritize teaching laboratory medicine to students in an efficient, timely manner [13].

The responses were shared with colleagues for collaborative learning and feedback purposes. Reflecting on their responses, the feedback was used to evaluate the effectiveness of the instructional strategies. The comments were evaluated to improve the methods and fill any knowledge gaps in subsequent classes. The feedback on the use of the OMP tool for assessing lessons learned during lectures revealed that, overall, 45.0% (n = 191) of students appreciated that it was conducted excellently, while another 32.0% (n = 136) rated it as a worthwhile initiative. It coincided with a study by Sahoo et al., in which 68% of students appreciated the method used [11].

Medical colleges around India confront significant challenges in adapting to CBME. The CBME curriculum in India has introduced many novel ideas, including a foundation course, early clinical exposure, and self-directed learning [14]. A self-directed learning environment differs significantly from a lecture-based classroom, where the instructor sets the objectives, administers the tests, and sets the pace for the course material. In the process of self-directed learning, the student establishes objectives, chooses how progress will be evaluated, establishes a schedule and the order of activities, finds resources, and solicits feedback [14,15]. Educators identified the need to clarify self-directed learning, tailor the curriculum to the basic sciences in interactive sessions, and provide early clinical exposure as crucial areas to focus on to implement CBME modules effectively. Appropriate teaching methodologies and efficient evaluation techniques improve competency acquisition [16]. The core of self-directed learning is students’ capacity to evaluate their learning needs, track their development, and actively control their learning strategies. This is promoted by the OMP, which asks students to actively reflect on their comprehension and learning processes while fostering self-control and metacognition.

Moreover, to comprehend the dynamics of learner autonomy and control in educational contexts, it is essential to recognize the differences between self-directed learning and self-regulated learning. Self-directed learning emphasizes students actively directing their learning, whereas self-regulated learning focuses on strategic planning and regulation [17,18]. Self-regulated learning is a desirable student quality that fosters a lifelong learning habit and is invaluable for aspiring health professionals. Self-regulated learning supports adult learners in organizing, carrying out, and assessing their learning objectives and results. The adult student increasingly takes control of their education rather than passively absorbing it, and it functions best as a supplement to traditional learning [18]. While formative and summative assessment feedback aims to close identified learning gaps, it remains a passive process, and student compliance is essential to its effectiveness.

Along with the summative assessment of learning focused on the attainment of outcomes, formative feedback-based assessment for learning and learner-centric assessment as learning are essential for facilitating cognitive and skill reinforcement [19]. OMP assessments help students develop self-regulated learning by encouraging rapid reflection on their comprehension and learning processes, as well as active management of their learning strategies. By encouraging students to engage with the content rather than merely listen, the exercise improves overall learning outcomes.

OMP can be applied in various large group settings with minor modifications. Recent studies on the multidimensional model of the OMP have identified three fundamental constructs, namely, connections, functions, and environmental context, to be responsible for value generation. The OMP can support learning and teaching in a post-COVID-19 environment, given its greater emphasis on online instruction [20]. Studies also documented that the modified OMP technique improved students’ metacognitive skills, which included their capacity to plan, monitor, assess, and re-plan, as well as their motivation to study. Students became more behaviorally proactive in their learning processes, particularly in time management [21]. In the post-COVID-19 era, where online/virtual teaching has gained importance, the instructor can use quick assessment tools to customize the learning process and, as necessary, support student-centered learning [21-23].

With many advantages for both students and teachers, the OMP is a flexible and efficient instrument for ongoing internal evaluation. Through encouraging active participation, offering prompt feedback, and facilitating adaptive instruction, OMP greatly improves the learning environment. When carefully implemented and with potential difficulties considered, OMP can transform the subtleties of teaching and learning in the classroom. As such, it is a promising part of present-day education.

Limitations

Despite the tool being incredibly straightforward and efficient, the present study did not collect follow-up data to address the challenging, unresolved questions. Hence, it was not possible to evaluate the students’ level of learning. The lack of a control group or a pre-post-test design is another limitation of the study. The single-institution setting and the study’s short duration limit its external validity. Convenience sampling was used to select the participants, which may introduce selection bias and limit generalizability. Moreover, the evidence in the current study is based on self-reported data rather than objective performance outcomes, and the responses may be subject to social desirability bias. Future studies in multi-institutional settings, with longitudinal assessment and inclusion of objective performance data, could help gauge the utility of the OMP for assessing academic achievement and its adaptability across various learning environments.

## Conclusions

Most participants agreed that the OMP was an excellent tool for measuring lessons learned during lectures. The OMP was valuable for evaluating the effectiveness of the concepts taught and for providing educators with information on the impact of their teaching strategies. The input provided in prior sessions helped them identify knowledge gaps and improve procedures. Data from individual sessions showed that continuous evaluation with the OMP enabled teachers to make modifications based on student feedback at the end of each lesson, thereby improving subsequent sessions. However, OMP, as a new approach, may temporarily boost engagement, and student responses might also be influenced by social desirability bias. The OMP shows potential as an effective tool based on students’ perceptions and functions as a learning catalyst that benefits both teachers and students by encouraging introspection, enhancing communication, and reaffirming the collaborative nature of education. Due to its ease of use, adaptability, and instantaneous impact, it is a promising component of modern assessment techniques.

## Appendices

Study questionnaire

Initial Information

1. Date

2. Name of the topic taken

3. The competency taken

4. Roll No

5. Gender

The seven questions were:

1. Please rate the overall usefulness of the session: 1.   2.   3.   4.   5.

(1 No usefulness.   5 Very high usefulness)

2. Whether all the learning objectives covered in today’s class were explained to you clearly

o Yes

o No

3. Whether the topic covered today was effective and enhanced your learning

o Yes

o No

4. If Yes, what did the faculty do today that was effective and enhanced your learning

i. Interactive session

ii. Group activity

iii. Audiovisual

iv. Feedback

v. Style of presentation

vi. No change

vii. Others ______________________

5. If No, what can the faculty do to improve the effectiveness of the class and therefore enhance your learning

i. Increase the interactive session

ii. Increase the group activity

iii. Increase audiovisual content

iv. More feedback

v. Improve the style of presentation

vi. No change required

vii. Others _________________________

6. What are the most important things you learnt today from the lecture?

7. What questions do you still have in your mind? Specify.
